# An Adaptive Model Filtering Algorithm Based on Grubbs Test in Federated Learning

**DOI:** 10.3390/e25050715

**Published:** 2023-04-26

**Authors:** Wenbin Yao, Bangli Pan, Yingying Hou, Xiaoyong Li, Yamei Xia

**Affiliations:** 1 School of Computer Science, Beijing University of Posts and Telecommunications, Beijing 100876, China; 2 Beijing Key Laboratory of Intelligent Telecommunications Software and Multimedia, Beijing University of Posts and Telecommunications, Beijing 100876, China; 3 School of Cyberspace Security, Beijing University of Posts and Telecommunications, Beijing 100876, China

**Keywords:** federated learning, byzantine-robust, poison attack defense, non-IID

## Abstract

Federated learning has been popular for its ability to train centralized models while protecting clients’ data privacy. However, federated learning is highly susceptible to poisoning attacks, which can result in a decrease in model performance or even make it unusable. Most existing defense methods against poisoning attacks cannot achieve a good trade-off between robustness and training efficiency, especially on non-IID data. Therefore, this paper proposes an adaptive model filtering algorithm based on the Grubbs test in federated learning (FedGaf), which can achieve great trade-offs between robustness and efficiency against poisoning attacks. To achieve a trade-off between system robustness and efficiency, multiple child adaptive model filtering algorithms have been designed. Meanwhile, a dynamic decision mechanism based on global model accuracy is proposed to reduce additional computational costs. Finally, a global model weighted aggregation method is incorporated, which improves the convergence speed of the model. Experimental results on both IID and non-IID data show that FedGaf outperforms other Byzantine-robust aggregation rules in defending against various attack methods.

## 1. Introduction

In recent years, federated learning has emerged as a novel paradigm for training machine learning models in a distributed environment. Data is stored on edge servers, IoT devices, mobile phones, personal computers, and other user devices, enabling users to perform distributed training on their own devices. The parameter server does not have direct access to user data, but aggregates models trained by user devices to obtain a global model [1]. Federated learning utilizes the data resources and computing power of edge devices while ensuring data privacy, making it possible to apply artificial intelligence technology in data-sensitive fields [2]. Consequently, federated learning has gained rapid momentum in recent years and is being widely adopted across industries [3,4]. For instance, federated learning can help solve the bottleneck problem in the pharmaceutical industry, where various companies find it difficult to cooperate to train a machine learning model due to privacy and security concerns [5].

Federated learning assumes that clients voluntarily participate in training and upload model parameters truthfully to the parameter server [6]. However, in practical applications, there may be malicious clients who upload misleading model parameters in federated learning, thereby compromising the accuracy of the global model. Such attacks are known as poisoning attacks [7,8], and the most common ways of carrying out such attacks are through sign-flipping and label-flipping [9,10]. In poisoning attacks, the presence of even a small number of malicious clients can significantly impact the accuracy of the global model. Therefore, it is crucial to develop effective defense mechanisms against these attacks.

There are currently several Byzantine-robust defense methods that analyze differences between models, such as Krum [11], multi-Krum, Coordinate-wise Median [12], etc. However, most of these methods may be weakened by or even ineffective on non-IID data. This is because machine learning is a data-driven technology, and the quality and distribution of the data greatly affects model training. Models with the same structure trained on similar training data often achieve similar target models. Measuring their similarity using methods such as Euclidean distance or cosine similarity can yield good results. Most current defense methods based on model similarity often rely on the high similarity between benign clients to achieve local model filtering and aggregation. Non-IID data refers to data that is not independent and identically distributed; it implied that the proportion of different classes of samples in each client’s local training dataset is different. In this case, structurally similar models trained on training datasets with different distributions will have reduced similarity between the resulting target models, and the extent of this reduction is related to the heterogeneity of the training data. Therefore, the heterogeneity of local training data between clients reduces the similarity between benign models, making it more difficult to distinguish between malicious and benign clients. Moreover, the trade-off between robustness and efficiency becomes more important when the data is non-IID. At this point, the accuracy of local models for classes with less training data may be relatively low. Federated learning thus requires the aggregation of multiple client models to ensure model convergence and training efficiency [13]. Therefore, in defending against poisoning attacks on non-IID data, if the parameter server aggregates too few local models to pursue robustness, it may slow down model convergence or even affect model accuracy. On the other hand, if the aim is to prioritize the efficiency of federated learning model training, this could result in a reduction in the robustness of the algorithm, as evidenced by a decrease in the success rate of defending against poisoning attacks.

This paper proposes an adaptive model filtering algorithm based on the Grubbs test in federated learning (FedGaf), which is a Byzantine-robust defense algorithm suitable for non-IID data. FedGaf implements dynamic poisoning attack defense by utilizing different metrics between client models while ensuring user privacy. It can defend against different poisoning attacks on both IID and non-IID data while maintaining a high convergence rate. Firstly, this work analyzes the performance of cosine similarity and Euclidean distance between local models at different stages under different attack scenarios. Based on this analysis, multiple adaptive model filtering algorithms are designed to defend against various types of poisoning attacks. As the core algorithm of FedGaf, the algorithm filters different numbers of local models each round to balance robustness and efficiency. Secondly, a dynamic decision-making mechanism is designed to select different model filtering algorithms for model filtering and generate candidate models based on the current performance of the global model. To protect user privacy, all candidate models are evaluated by clients, and the global model is determined based on client reports. Finally, to improve the influence of high-quality models on the global model and speed up convergence, model weights are determined based on model similarity. The experimental results on different poisoning attack methods using the MNIST dataset show that FedGaf achieves a better trade-off between robustness and efficiency in the majority of scenarios, outperforming Krum and Multi-Krum.

## 2. Related Work

The concept of federated learning was first proposed by McMahan when he introduced FedSGD, which performs one round of stochastic gradient descent (SGD) on a local model on each client and uploads the model to the parameter server for aggregation. While FedSGD addresses the challenge of sensitive data privacy and achieves the same accuracy as traditional centralized model training on IID data, frequent model uploads and distribution significantly increase the communication burden, resulting in efficiency issues. In 2017, McMahan et al. proposed an improved algorithm, Federated Averaging (FedAvg) [14], which allows clients to perform multiple rounds of SGD before uploading their models to the parameter server for aggregation. This approach effectively reduces communication rounds and improves the efficiency of federated learning, making it the most classic algorithm in the field of federated learning.

Research has found that existing federated learning systems are vulnerable to various attacks [15,16]. The most common form of interference by malicious participants is to upload misleading model update information, which can impede the convergence of the global model and is known as a poisoning attack. If a Federated Learning system cannot defend against such attacks, it may cause the model’s performance to decline or become unusable, providing incorrect results to users, and could even lead to serious accidents. Poisoning attacks in Federated Learning can be classified as targeted attacks [17] and untargeted attacks based on the attacker’s objective.

The primary goal of targeted attacks is to make the model predict specific samples as another specified category. In contrast, the goal of untargeted attacks is to reduce the global accuracy of the model or even make the global model unusable. The objective of the adversary is arbitrary, as long as they can reduce the accuracy of the model and provide incorrect predictions [18]. Since untargeted attacks are relatively simple to implement and have a significant impact on the performance of the global model, this paper focuses primarily on untargeted attacks.

Untargeted attacks mainly include label-flipping attacks and sign-flipping attacks. Label-flipping is a data poisoning attack, in which a malicious client participating in training flips the labels of the locally saved dataset before training the local model. Sign-flipping is a model poisoning attack, in which a malicious client trains the local model using the same method as other clients, but reverses the model gradient when uploading it to the parameter server, thus uploading incorrect model parameters to the parameter server.

To defend against poisoning attacks in federated learning, some works have researched defense mechanisms [19,20]. Krum, Multi-Krum, and Coordinate-wise Median are representative defense algorithms against poisoning attacks on IID data. In Krum, the parameter server calculates the Euclidean distance between each local model in each round, where $d_{i,j}=\|g_{i}-g_{j}\|{}^{2}\left(i\neq j\right),$ and selects the n−f−1 smallest distances for each local model, accumulating them as the score for that gradient, where n is the number of local models and f is the number of malicious models. After calculating the scores for all gradients, the one with the lowest score is selected as the global model. In Multi-Krum, after Krum is executed, the gradient with the lowest score is selected and removed from all gradients, and then the scores for each gradient are recalculated until m gradients are selected. The final global model is the average of the m selected gradients. In the Coordinate-wise Median algorithm, the parameter server computes the median of each model parameter as the parameter for the corresponding position of the global model.

Some studies have focused on defending against poisoning attacks on non-IID data, as many defense strategies may fail in such situations. One approach is to use the Shapley value as an indicator of a participant’s contribution to federated learning and identify malicious participants [21,22], but the computation cost of the Shapley value is huge. Li et al. proposed that poison attacks may be associated with witch attacks and indirectly detected through the detection of witch attacks [23], but this approach has limited practicality. Bonawitz et al. reduced the external variance of model update information caused by non-IID to mitigate the effect of poison attacks [24], but this also reduced the convergence speed of the global model. Zhai et al. suggested that participants publicly disclose a portion of their training data before the start of the task to serve as prior knowledge for detecting poison attacks [25], but this sacrifices user privacy. To achieve an efficient and reliable defense method for poisoning attacks that is applicable to non-IID data while protecting user privacy, this paper explores this problem and proposes the FedGaf. Unlike the above work, FedGaf still uses Euclidean distance and cosine similarity to filter models based on model similarity, which brings lower additional computation costs for federated learning compared to using Shapley values, and does not have a significant impact on the training efficiency of federated learning. In addition, experimental results show that FedGaf can still maintain its defensive performance in multiple different attack scenarios, directly and effectively achieving defense against poisoning attacks, with broad applicability. Finally, FedGaf strictly adheres to the principle of protecting user data privacy in federated learning and does not intervene in user data, let alone collect private data from users.

## 3. Design of FedGaf

In poisoning attacks, whether they are data poisoning attacks or model poisoning attacks, the ultimate goal is to affect the global model by influencing the model parameters uploaded to the parameter server. Therefore, most existing work focuses on filtering out malicious models or reducing the impact of malicious models on the global model by analyzing the relationships between local model parameters. However, most of these works only focus on a certain specific metric among the models or retain a fixed number of clients during model filtering, which leads to insufficient system robustness or a decrease in model convergence speed. To address this issue, we propose a Byzantine-robust defense algorithm with dynamic decision-making and adaptive filtering called FedGaf. FedGaf takes user privacy as a fundamental principle, achieves defense against various poisoning attacks, and maintains robustness and efficiency when the local data is non-IID. Compared to traditional federated learning systems, this algorithm requires clients to independently maintain local test data and adds an evaluation module to evaluate the performance of the model on local test data and report it to the parameter server. Correspondingly, the parameter server has a filtering module that receives model performance reports from clients and determines the global model accordingly.

**Figure 1:**
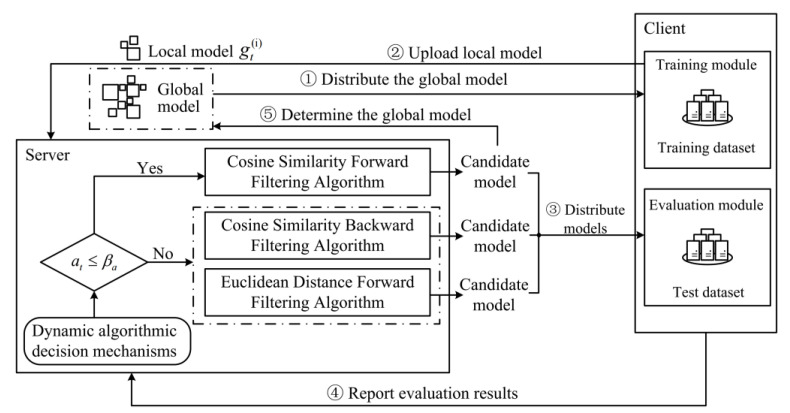
Structural design of FedGaf.

As shown in Figure 1, at the beginning of each round of communication, the server distributes the global model to the clients who participate in the training. Then, the clients train their local models using their local training data and upload their local models to the parameter server after training. After collecting all the local models from the clients, the server automatically selects filtering algorithms based on the strategy conversion threshold $\beta_{a}$ and the accuracy of the previous global model, denoted as $a_{t}$ in FedGaf. If $a_{t}\leq \;\beta_{a}$, the cosine similarity forward filtering algorithm will be selected. Otherwise, the cosine similarity backward filtering algorithm and Euclidean distance forward filtering algorithm will be selected. The selected filtering algorithms filter the local models and aggregate them to obtain their respective candidate models. These candidate models are distributed to all clients for model evaluation. The clients evaluate the accuracy of every candidate model using their local test data and upload the results to the server. Finally, the server combines all the client evaluation results to determine the global model for the current round from candidate models and update the accuracy $a_{t}$.

### 3.1. Adaptive Filtering Algorithm Based on Grubbs Test

**Figure 2:**
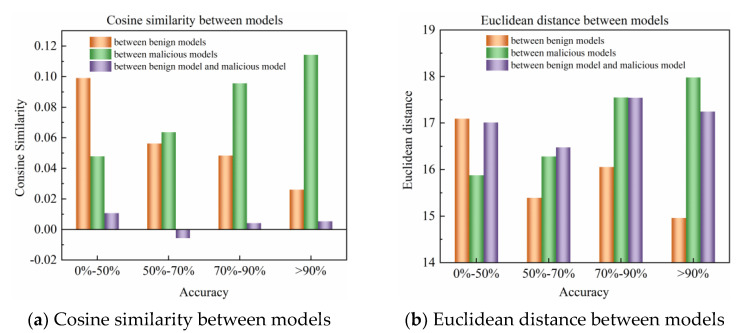
Changes in cosine similarity and Euclidean distance between models under 33% client’s label-flipping attack.

To investigate the defense against different poison attacks, we simulated multiple poison attack scenarios in federated learning. We studied the performance of cosine similarity and Euclidean distance, two similarity evaluation metrics, in the model training process. Figure 2 shows the data for training the MLP model on a non-IID distributed MNIST dataset under 33% client’s label-flip attack. Figure 2a shows the performance of cosine similarity among models at different accuracy intervals during model training. It can be observed that as the model accuracy increases, the cosine similarity between benign models decreases continuously, while that between malicious models increases continuously, resulting in a significant decrease in the difference between them. However, the cosine similarity between benign and malicious models is not significantly affected by model accuracy. Figure 2b shows the performance of Euclidean distance among models at different accuracy intervals during model training. The experimental results indicate that as the model accuracy increases, the Euclidean distance between benign models generally decreases, while that between malicious models generally increases. After analyzing multiple attacks using a similar method, we found that different attacks may lead to different results. However, there are still commonalities among benign models that can be utilized: (1) when the accuracy is low, the cosine similarity between benign models is always at a higher level; (2) when the accuracy is high, the Euclidean distance between benign models is always at a lower level.

The change pattern of model similarity indicates the feasibility of model filtering methods based on model similarity, and there are currently some poisoning attacks defense methods based on model similarity, such as Krum and Multi-Krum. However, they both have some problems. Krum selects only one local model as the global model in each round, which inevitably leads to a significant decrease in training efficiency. On the other hand, Multi-Krum’s strategy of aggregating a fixed number of local models in each round sacrifices robustness. For example, assume that there are 30% malicious models in the federated learning system, and 10 clients are randomly selected for training in each round. Here, Multi-Krum assumes that 3 malicious clients participate in training in each round, and chooses to aggregate 7 local models in each round for the sake of training efficiency. However, due to the random selection of clients in federated learning, it is possible to select 4 or even more malicious clients in a round of training, and Multi-Krum will inevitably aggregate malicious models, which will affect the global model. To avoid this situation, an adaptive model filtering algorithm that does not fix the number of aggregated models is needed. Therefore, based on the Grubbs test [26], this paper proposes an adaptive filtering algorithm. The Grubbs test is a statistical method for outlier detection based on the ratio of the difference between the test data and the sample mean to the sample standard deviation. Compared to the model filtering methods with a fixed number of filters per round, such as Krum and Multi-Krum, this algorithm adjusts the number of models to be retained adaptively based on the actual performance of local models in each round. By adjusting the strict filtering coefficient gamma, different trade-offs between robustness and efficiency can be achieved. A larger gamma is typically used to obtain higher efficiency, while a smaller gamma is used to achieve stronger robustness. In FedGaf, three adaptive filtering algorithms are provided: cosine similarity forward filtering, cosine similarity backward filtering, and Euclidean distance forward filtering.

#### 3.1.1. Cosine Similarity Forward Filtering

Based on the trends in cosine similarity between benign models, we have designed a cosine similarity forward filtering algorithm. In the t-th communication round, the parameter server collects the local models $g_{t}^{\left(i\right)}$ from all participating clients, where $i\in C_{t}$ and $C_{t}$ is the set of clients participating in this round. The server then calculates the cosine similarity between all pairs of local models to obtain the similarity matrix S. Specifically, for $\forall i,j\in C_{t},\;S_{i,j}=\frac{g_{t}^{\left(i\right)}\cdot g_{t}^{\left(j\right)}}{\left\|g_{t}^{\left(i\right)}\|\|g_{t}^{\left(j\right)}\right\|}$. After calculating the cosine similarity matrix, the cosine similarity forward filtering algorithm is applied to filter the local models based on this matrix. The specific steps are as follows.

**Step 1.** For local model $g_{t}^{\left(i\right)}$, select $\left|C_{t}\right|-f-1$ largest cosine similarities with other models from $\left\{S_{i,j}|j\in C_{t},i\neq j\right\}$, denoted as $\left\{s_{i,1},s_{i,2},\ldots ,s_{i,\left|C_{t}\right|-f-1}\right\}$, where $\left|C_{t}\right|$ is the number of clients participating training and f is the estimated number of malicious models.

**Step2.** The scores of each local model are obtained by summing up the largest $\left|C_{t}\right|-f-1$ cosine similarities for each local model, and all the final score is obtained as $CS=\{CS_{i}|i\in C_{t}\},$ where $CS_{i}=\overset{\left|C_{t}\right|-f-1}{\underset{k=1}{\sum }}s_{i,k}$.

**Step 3.** Calculate the mean and standard deviation of CS, denoted as $\bar{CS}$ and $\sigma_{CS}$, respectively, which can be expressed as follows:

(1) $$\bar{CS}=\frac{\sum_{i\in C_{t}}CS_{i}}{\left|C_{t}\right|}$$

(2) $$\sigma_{CS}=\sqrt{\frac{\sum_{i\in C_{t}}{\left(CS_{i}-\bar{CS}\right)}^{2}}{\left|C_{t}\right|}}$$

**Step 4.** Calculate the filtering index $Gb_{i}$ for each local model $g_{t}^{\left(i\right)}$ as $Gb_{i}=\frac{CS_{i}-\bar{CS}}{\sigma_{CS}}$. If $Gb_{i}<-\gamma$, the local model $g_{t}^{\left(i\right)}$ is classified as malicious and $CS_{i}$ is removed from CS, where γ is the strict filtering coefficient. If any local model is filtered out, return to **Step 3** and repeat the process until no models are filtered out.

#### 3.1.2. Cosine Similarity Backward Filtering

Due to the decrease of cosine similarity between benign models as the accuracy increases, we have designed a cosine similarity backward filtering algorithm. This algorithm first uses the same calculation method to obtain the cosine similarity matrix S, and then performs the following filtering process.

**Step 1.** For each local model $g_{t}^{\left(i\right)}$, select f largest cosine similarities with other models from $\left\{S_{i,j}|j\in C_{t},i\neq j\right\}$, denoted as $\left\{s_{i,1},s_{i,2},\ldots ,s_{i,f}\right\}$, where f is the estimated number of malicious models.

**Step 2.** Calculate the sum to obtain $CR=\{CR_{i}|i\in C_{t}\}$, where $CR_{i}=\overset{f}{\underset{k=1}{\sum }}s_{i,k}$.

**Step 3.** Calculate the mean and standard deviation of CR, obtain $\bar{CR}$ and $\sigma_{CR}$.

**Step 4.** Calculate the filtering index $Gb_{i}$ for each local model $g_{t}^{\left(i\right)}$ as $Gb_{i}=\frac{CR_{i}-\bar{CR}}{\sigma_{CR}}$. Use $Gb_{i}>\gamma$ as the criterion for detecting malicious models. If there are malicious models detected, remove them and return to **Step 3.** Repeat this process until no malicious models can be detected.

#### 3.1.3. Euclidean Distance Forward Filtering

As high-accuracy models usually have small Euclidean distances between benign models, we have designed a Euclidean distance forward filtering algorithm based on Euclidean distance in addition to the cosine similarity-based filtering algorithm. In this algorithm, the server collects the local models $g_{t}^{\left(i\right)}$ and calculates the Euclidean distance between them to obtain the matrix *S*, where $\forall i,j\in C_{t},\;S_{i,j}=S_{i,j}=\|g_{t}^{\left(i\right)}-g_{t}^{\left(j\right)}\|$. After the matrix calculation is completed, Euclidean distance forward filtering is performed with the following procedure.

**Step 1.** For each local model $g_{t}^{\left(i\right)}$, select $\left|C_{t}\right|-f-1$ largest distances with other models from $\left\{S_{i,j}|j\in C_{t},i\neq j\right\}$, denoted as $\left\{s_{i,1},s_{i,2},\ldots ,s_{i,\left|C_{t}\right|-f-1}\right\}$, where $\left|C_{t}\right|$ is the number of clients participating training and f is the estimated number of malicious models.

**Step 2.** Sum the selected Euclidean distances for each local model $g_{t}^{\left(i\right)}$ to obtain $ED=\{ED_{i}|i\in C_{t}\}$, where $ED_{i}=\overset{\left|C_{t}\right|-f-1}{\underset{k=1}{\sum }}s_{i,k}$.

**Step 3.** Calculate the mean and standard deviation of ED, obtain $\bar{ED}$ and $\sigma_{ED}$.

**Step 4.** Compute the filter index $Gb_{i}$ for each local model $g_{t}^{\left(i\right)}$ as $Gb_{i}=\frac{ED_{i}-\bar{ED}}{\sigma_{ED}}$. Use $Gb_{i}>\gamma$ as the criterion for detecting malicious models. If there exist any malicious models, remove them and go back to **Step 3.** until no malicious models can be detected.

#### 3.1.4. Model Weighted Aggregation

To accelerate the convergence of the global model, FedGaf has designed corresponding methods for calculating model weights for each filtering algorithm to enhance the influence of high-quality models during global model aggregation. At the same time, it can reduce the weight of malicious clients, so as to improve the quality of defense and speed up the convergence of the model.

In the cosine similarity forward filtering algorithm, models with higher scores are considered to be of higher quality, and the cosine similarity ranges from [−1, 1]. In order to avoid using a negative number as the weight for model aggregation, it is necessary to convert $CS_{i}$ into $e^{CS_{i}}$, so that the weight of the model becomes a positive number and the relative size relationship between different model weights is maintained. Therefore, the weights of the models retained by the cosine similarity forward filtering algorithm are calculated using the following formula.

(3) $$w_{i}=\frac{e^{CS_{i}}\cdot \left|D^{\left(i\right)}\right|}{\sum_{k\in C_{a}}e^{CS_{k}}\cdot \left|D^{\left(k\right)}\right|}$$

where $i\in C_{a}\subseteq C_{t}$, $C_{a}$ represents the filtered set of local models, and $\left|D^{\left(i\right)}\right|$ represents the size of the corresponding client’s local training set.

The models with lower scores obtained from the cosine similarity reverse filtering algorithm and the Euclidean distance forward filtering algorithm are considered to be of higher quality. Therefore, the models retained by the cosine similarity reverse filtering algorithm and the Euclidean distance forward filtering algorithm respectively use the following formulas to calculate the weights.

(4) $$w_{i}=\frac{\frac{\left|D^{\left(i\right)}\right|}{e^{CR_{i}}}}{\sum_{k\in C_{a}}\frac{\left|D^{\left(k\right)}\right|}{e^{CR_{k}}}}$$

(5) $$w_{i}=\frac{\frac{\left|D^{\left(i\right)}\right|}{ED_{i}}}{\sum_{k\in C_{a}}\frac{\left|D^{\left(k\right)}\right|}{ED_{k}}}$$

Note that since the cosine similarity can take negative values, $CR_{i}$ needs to be converted to $e^{CR_{i}}$ to calculate the model weight, while the Euclidean distance is always positive, so $ED_{i}$ can be directly used as the basis for calculating the model weight.

Using the adaptive filtering algorithm and the corresponding weight calculation method, each filtering algorithm can aggregate local models using $g_{t}=\underset{i\in C_{a}}{\sum }w_{i}\cdot g_{t}^{\left(i\right)}$ to generate a candidate model. However, generating three candidate models per communication round is not only time-consuming on the server-side, but also imposes a computational burden on the clients, since the candidate models need to be validated and evaluated by the clients. Therefore, FedGaf introduces a dynamic algorithmic decision mechanism to dynamically activate the above filtering algorithm to reduce the computational cost as much as possible.

### 3.2. Dynamic Algorithmic Decision Mechanism

To implement a dynamic filtering algorithmic decision mechanism, the server in FedGaf maintains a global model accuracy $a_{t}$, which represents the performance of the global model in the client testing set for round t ($a_{0}=0$). After collecting the required local models, the server compares $a_{t}$ with the pre-set strategy conversion threshold $\beta_{a}$. If $a_{t}\leq \beta_{a}$, it indicates that the global model accuracy is poor, and benign models with high cosine similarity can be preserved. Therefore, FedGaf uses the cosine similarity forward filtering algorithm to filter the local models and generate a candidate model. If $a_{t}>\beta_{a}$, it indicates that the global model accuracy is good, and the cosine similarity and Euclidean distance between benign models are both low. In this case, the server uses the cosine similarity backward filtering algorithm and the Euclidean distance forward filtering algorithm respectively to filter the local models and generate candidate global models.

To determine the final global model and update $a_{t}$, FedGaf needs to evaluate the performance of each candidate model. To protect user data privacy, the client datasets in FedGaf are not visible to the server. However, considering the possibility that the server may not have available testing sets, FedGaf uses client-side evaluation to verify model performance. In FedGaf, each client maintains a local training set and a local test set, where client i’s local training set is denoted as $D^{\left(i\right)}$ and the local test set is denoted as $D_{0}^{\left(i\right)}$. The client needs to evaluate the performance of the candidate models on the local test set and return the accuracy $\alpha^{\left(i\right)}$ and $\left|D_{0}^{\left(i\right)}\right|$ to the server. After receiving all the reports, the server calculates the candidate model accuracy α as follows:

(6) $$\alpha =\frac{\sum_{i\in C}\alpha^{\left(i\right)}\left|D_{0}^{\left(i\right)}\right|}{\sum_{i\in C}\left|D_{0}^{\left(i\right)}\right|}$$

where C is the set of all clients in the FL system. If there are multiple candidate models, the server selects the one with higher accuracy as the global model for t-th round and updates $a_{t}$ with its accuracy. Note that the calculation of the candidate model accuracy depends on the local test sets of all clients, which can be tampered with by malicious clients and lead to imprecision in $a_{t}$. However, since FedGaf only needs $a_{t}$ to make a rough judgment of model performance, it is not affected by tampering with local test sets under the assumption that most clients are honest.

## 4. Performance Evaluation

To evaluate the ability of FedGaf to resist different poison attacks in different scenarios, this paper implements FedGaf based on Pytorch and implements Krum and Multi-Krum, two Byzantine-robust methods, for comparison. We used three poisoning attack methods, including the label-flipping attack, sign-flipping attack, and random-label attack based on label-flipping modifications, on the MNIST and CIFAR-10 datasets to evaluate FedGaf’s performance and experiments are conducted at different attack rates. The large number of experimental results show that, under strict protection of user data privacy, FedGaf can achieve better defense in both IID and non-IID data.

On IID MNIST data, FedGaf can achieve up to 95.47% and 95.43% accuracy against the label-flipping attack and sign-flipping attack, respectively, with the FL baseline at 96.08%, which is close to the baseline. Krum has 93.31% and 94.01%, and Multi-Krum has 95.86% and 95.93% accuracy against the two attacks, respectively. Both FedGaf and the two comparison algorithms can maintain good stability under these two attacks, but FedGaf and Multi-Krum are better than Krum. In random-label attack, which has a smaller impact on the global model, Krum and Multi-Krum algorithms lose their robustness and cannot work properly, with accuracy at only around 10%. However, FedGaf achieves 94.94% accuracy while maintaining robustness. On non-IID data, FedGaf achieves 94.54% and 94.90% accuracy against the label-flipping attack and sign-flipping attack, with the FL baseline at 94.83%, which is close to the baseline. Krum has 89.86% and 90.92%, and Multi-Krum has 94.50% and 94.67% accuracy against the two attacks, respectively. At this point, FedGaf’s accuracy is significantly higher than Krum and slightly higher than Multi-Krum, but FedGaf has significantly better stability than Multi-Krum. In random-label attacks, Krum and Multi-Krum still cannot work, while FedGaf achieves 93.93% accuracy, with slightly reduced stability compared to the IID scenario.

In the CIFAR-10 dataset, due to the complexity of the selected model and the long training time, the model did not converge during the experiments. Therefore, in CIFAR-10, the advantage of FedGaf over Krum and Multi-Krum can be directly reflected in the convergence speed. A large number of experimental results show that FedGaf can achieve a convergence speed no weaker than the other two methods in various scenarios, and in most scenarios, FedGaf’s model convergence speed is significantly faster.

### 4.1. Experimental Setup

**Figure 3:**
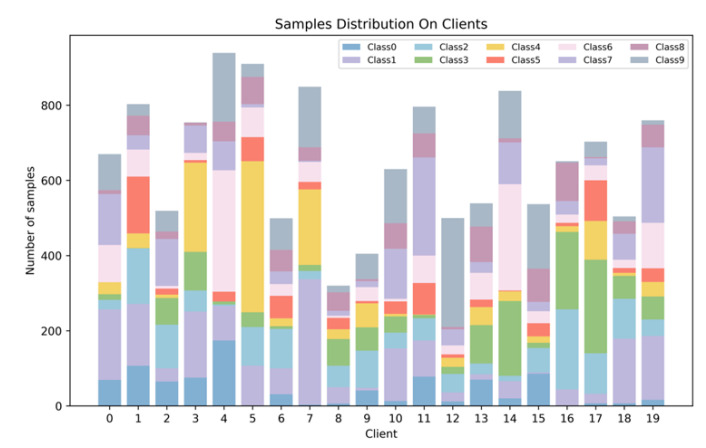
Distribution of various samples among clients.

Table 1 presents the main parameters involved in the experiments and their reference settings. In the experiment, we trained MLP model with two dense layers on the MNIST dataset and trained ResNet model on the CIFAR-10 dataset. FedGaf requires all clients to hold local test sets, so in the experiment, we took 10% of the test set and distributed it as the local test set to all clients. To simulate IID data, we randomly assigned the same number of samples from the training set to each client. As for non-IID data partitioning, we partitioned the training set into non-IID sets according to the Dirichlet distribution and distributed them to each client. This method can also ensure that the number of samples for different clients is different. The sample details on different clients are shown in Figure 3. To simulate attacks from clients, we set an attack rate $r_{attack}$, and after the data was partitioned, we selected $N\cdot r_{attack}$ clients as malicious clients and used them to launch attacks during training. In selecting the attack rate $r_{attack}$, we experimented with rates of 0.33 and 0.49. When studying poison attacks in federated learning, it is common to select about one-third of the clients as malicious clients, as in machine learning, when the proportion of abnormal data reaches a certain level, the model performance will show a significant decline. Our experiments show that the model performance in federated learning will be significantly affected and decrease when the attack rate is 0.33. Therefore, we chose 0.33 as one of the rates. In addition, federated learning is a clearly distributed system, and in most distributed systems, it is often assumed that 50% is an important threshold that affects the stability of the distributed system. This is particularly evident in blockchain, where if the proportion of malicious nodes in the system exceeds 50% and is controlled by an organization, the entire blockchain system will collapse. Similarly, federated learning is also a distributed system, and methods such as FedGaf, which are based on model similarity, are highly dependent on the proportion of malicious nodes. Therefore, we conducted experiments with an attack rate of approximately 50%, specifically 49%. Note that FedGaf can still operate when facing an attack rate exceeding 50%. More generally, when facing lower attack rates (e.g., 5% to 10%), a few malicious models have limited impact on the global model, and malicious models are easier to filter out in the adaptive filtering algorithm. Thus, it is possible to increase the strict filtering coefficient γ for more tolerant filtering, retaining more benign models and improving model performance. In contrast, when facing higher attack rates (exceeding 50%), stricter model filtering can be achieved by further reducing the strict filtering coefficient γ, filtering out malicious models as much as possible, and ensuring the robustness of the federated learning system.

### 4.2. Defending Label-Flipping Attack

**Figure 4:**
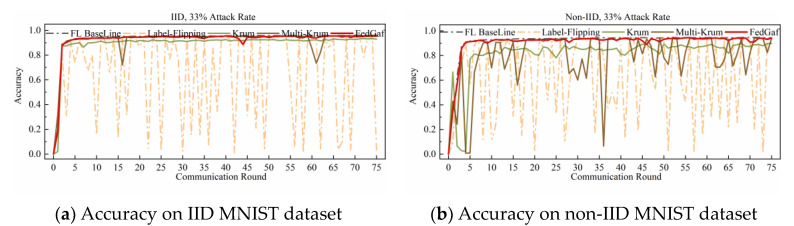
Accuracy under 33% label-flipping attack on MNIST dataset.

**Figure 5:**
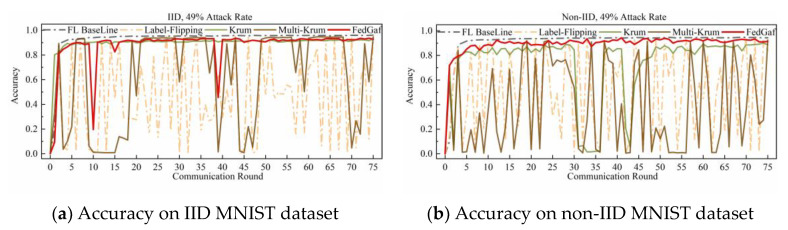
Accuracy under 49% label-flipping attack on MNIST dataset.

This paper implements a label-flipping attack by adding 2 to the labels of all samples of the malicious client and taking the modulus of the number of classes in the dataset. Figure 4 and Figure 5 show the performance of Krum, Multi-Krum, and FedGaf on MNIST dataset.

As shown in Figure 4a, the label-flip attack severely damages the FL system, and the performance of the three defense algorithms is not significantly different from the baseline, except that the accuracy of Krum is significantly lower than that of Multi-Krum and FedGaf. The peak performance of FedGaf is comparable to that of Multi-Krum, but it is evident that the accuracy of Multi-Krum decreases significantly in the 17th and 62nd rounds. This is because we only specify the proportion of malicious clients in the system initialization, which does not mean that only this proportion of malicious clients is selected for each communication round. This phenomenon reflects the disadvantage of defense algorithms with fixed aggregation quantities. Although it improves accuracy and convergence speed, it sacrifices some robustness. FedGaf, on the other hand, shows no significant fluctuations during training but can still maintain a similar accuracy performance to Multi-Krum. As shown in Figure 4b, on non-IID data, the gap is even more significant, with Krum’s algorithm showing significantly lower accuracy performance than the other two methods, and Multi-Krum showing significant fluctuations. Note that Multi-Krum selects three local models for aggregation per round, which is already an optimal choice, and selecting other quantities would lead to worse results. FedGaf performs well, achieving near-baseline accuracy while maintaining stable performance. Figure 5 presents the experimental results when facing a 49% label-flip attack. It is evident that FedGaf outperforms Krum and Multi-Krum in this scenario. As the number of attackers increases, Multi-Krum becomes highly unstable, with its defense against poison attacks frequently failing, resulting in a significant drop in accuracy. In contrast, FedGaf remains robust. From Figure 5b, it can be observed that although Krum still guarantees the security of the federated learning system, it still suffers from model performance issues on non-IID data. FedGaf, on the other hand, achieves significantly better model accuracy than Krum.

**Figure 6:**
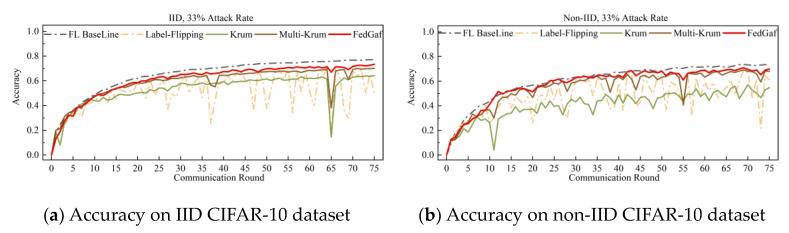
Accuracy under 33% label-flipping attack on CIFAR-10 dataset.

**Figure 7:**
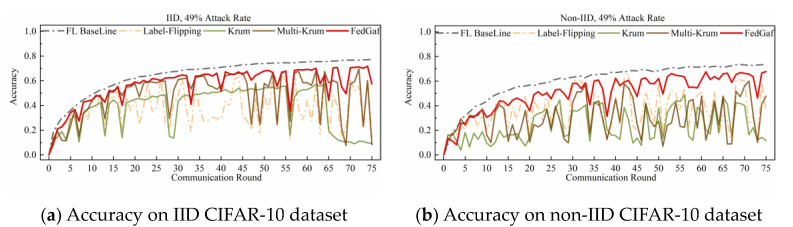
Accuracy under 49% label-flipping attack on CIFAR-10 dataset.

Figure 6 and Figure 7 show the performance on the CIFAR-10 dataset. Training a ResNet model on the CIFAR-10 dataset is significantly slower to converge than training an MLP model on the MNIST dataset, making FedGaf’s advantages more evident. On IID data, FedGaf’s model convergence is significantly faster than Krum’s and its stability is slightly better than Multi-Krum’s. On non-IID data, FedGaf outperforms Krum and Multi-Krum in both convergence speed and stability. Especially when the attack rate reaches 49%, as shown in Figure 7b, FedGaf has a clear advantage over the other two algorithms, with better stability and training efficiency than Krum and Multi-Krum.

Overall, in the context of defending against label-flipping attacks, FedGaf demonstrates superior robustness and performance in comparison to Krum and Multi-Krum. This is supported by its ability to maintain stable training efficiency even when facing high attack rates, indicating its efficacy in preventing the malicious influence of adversarial participants in a federated learning system.

### 4.3. Defending Sign-Flipping Attack

**Figure 8:**
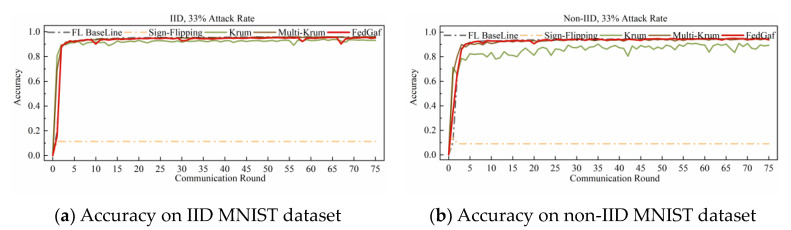
Accuracy under 33% sign-flipping attack on MNIST dataset.

**Figure 9:**
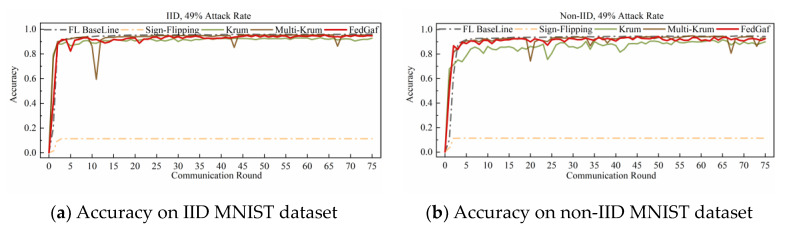
Accuracy under 49% sign-flipping attack on MNIST dataset.

This paper implements a sign-flipping attack by calculating the real gradient of the malicious client, multiplying it by a boosting factor of −4, and then recalculating the local model to increase its impact on the global model. Figure 8 and Figure 9 show the performance of Krum, Multi-Krum, and FedGaf on the MNIST dataset. The experimental results show that all three algorithms are very effective in defending against sign-flipping attacks on the MNIST dataset, and their model accuracies are quite good. Both FedGaf and Multi-Krum perform exceptionally well, achieving accuracy close to the baseline. However, Krum’s accuracy is slightly lower due to its characteristic of selecting only one local model, particularly on non-IID data. As for robustness, all three algorithms exhibit strong robustness in this scenario, with FedGaf showing slightly better stability.

**Figure 10:**
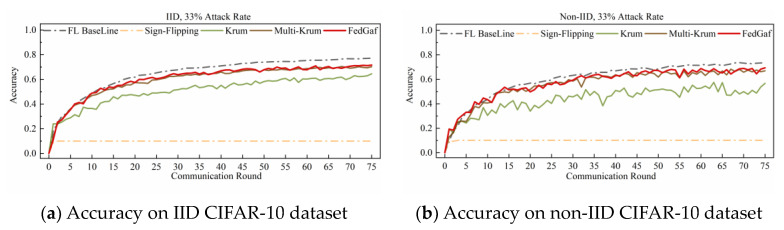
Accuracy under 33% sign-flipping attack on CIFAR-10 dataset.

**Figure 11:**
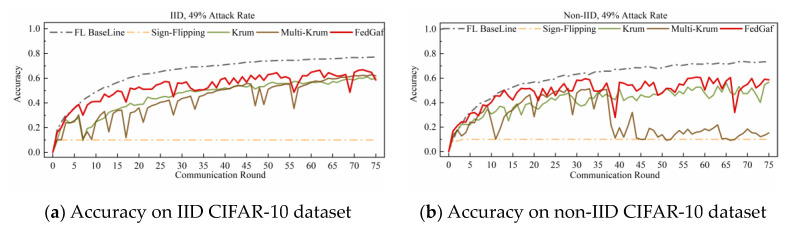
Accuracy under 49% sign-flipping attack on CIFAR-10 dataset.

The performance of various algorithms in defending against sign-flipping attacks on the CIFAR-10 dataset differs from that on the MNIST dataset. Figure 10 and Figure 11 show their performance on the CIFAR-10 dataset. As shown in Figure 10, when the attack rate is 33%, FedGaf and Multi-Krum perform better than Krum, with a significantly faster convergence rate and better robustness. However, when the attack rate increases to 49%, the performance of both Multi-Krum and FedGaf declines significantly. As shown in Figure 11a, Multi-Krum’s performance is now comparable to Krum, while FedGaf’s convergence rate and robustness have also declined, but even so, FedGaf’s performance is still better than that of the other algorithms. On non-IID data, Multi-Krum’s performance decline is particularly pronounced, losing robustness completely in the second half of training, while FedGaf shows several significant accuracy fluctuations, but its overall performance is still better than Krum, mainly manifested in significantly higher model accuracy than Krum.

In summary, FedGaf is superior to Krum and Multi-Krum algorithms in defending against sign-flipping attacks.

### 4.4. Defending Random-Label Attack

**Figure 12:**
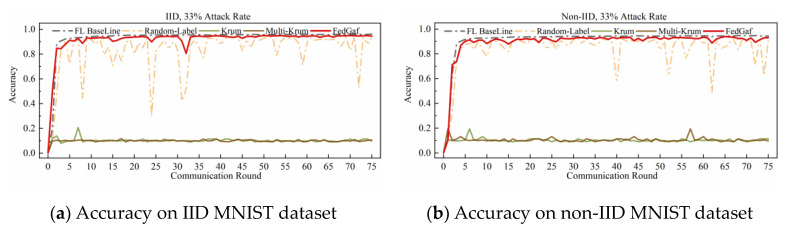
Accuracy under 33% random-label attack on MNIST dataset.

**Figure 13:**
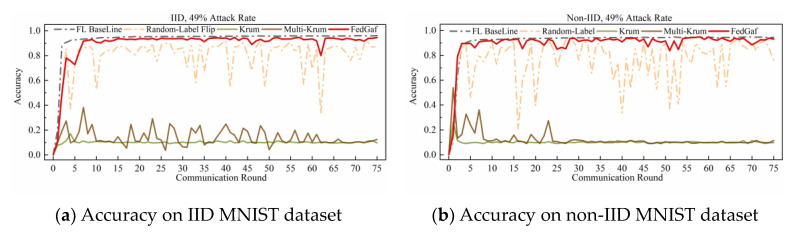
Accuracy under 49% random-label attack on MNIST dataset.

This paper implements a random-label attack by randomly assigning labels within the range of classes in the dataset to the samples of the malicious client. Figure 12 and Figure 13 show the training performance of Krum, Multi-Krum, and FedGaf on the MNIST dataset.

The experimental results demonstrate that this attack method is fatal to Krum and Multi-Krum algorithms on the MNIST dataset. In all scenarios, Krum and Multi-Krum are unable to function normally and maintain an accuracy rate of around 10% when facing random-label attacks. In contrast, FedGaf can still ensure the convergence of the global model, maintaining a high model accuracy rate and better robustness in all scenarios. FedGaf has a significant advantage over Krum and Multi-Krum in defending against random-label attacks on the MNIST dataset.

**Figure 14:**
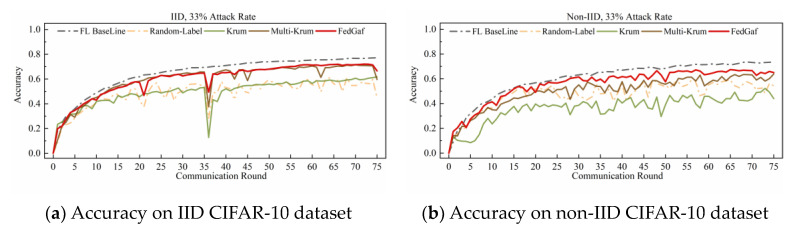
Accuracy under 33% random-label attack on CIFAR-10 dataset.

**Figure 15:**
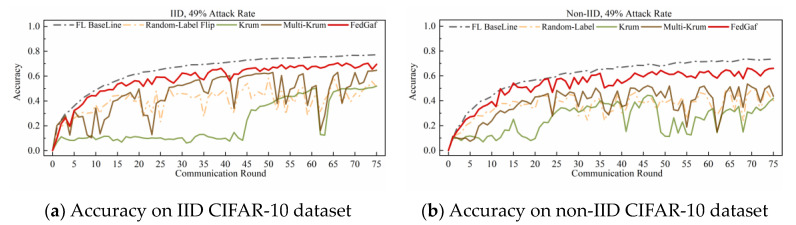
Accuracy under 49% random-label attack on CIFAR-10 dataset.

As shown in Figure 14 and Figure 15, random-label attacks on the CIFAR-10 dataset do not have a devastating impact on Krum and Multi-Krum algorithms, but FedGaf still has a significant advantage. As shown in Figure 14a, when the attack rate is 33% on IID data, FedGaf’s performance is close to Multi-Krum and significantly better than Krum. On non-IID data, as shown in Figure 14b, FedGaf’s performance is significantly better than Multi-Krum and Krum. When the attack rate increases to 49%, FedGaf’s advantage becomes even more pronounced. As shown in Figure 15, FedGaf achieves much better convergence speed and stability than the other two algorithms on both IID and non-IID data.

In conclusion, FedGaf has a significant advantage over Krum and Multi-Krum algorithms in defending against random-label attacks.

## 5. Conclusions

This paper proposes a Byzantine-robust defense algorithm, FedGaf, based on adaptive filtering. Unlike other model-based analysis algorithms, FedGaf fully utilizes the trends of cosine similarity and Euclidean distance among models during global model training. It integrates multiple effective local model adaptive filtering algorithms and dynamically selects filtering algorithms based on the global model performance, achieving superior Byzantine robustness with minimal additional computational overhead while maintaining training efficiency. Experimental results on MNIST dataset and CIFAR-10 dataset demonstrate that FedGaf outperforms Krum and Multi-Krum in terms of the overall performance of robustness and efficiency.

## Figures and Tables

**Table 1:** Reference setting of experiment.

Symbol	Description	Setting
T	Communication rounds	75
B	Local batch-size	10
E	Local training epochs	5
N	Number of clients	100
c	Percentage of clients participating in training	0.1
α	Dirichlet distribution parameter	1.0
$r_{attack}$	Proportion of malicious clients	0.33 or 0.49
γ	Strict filtering coefficient	1.1–1.3
$\beta_{a}$	Strategy conversion threshold	0.4

## Data Availability

http://yann.lecun.com/exdb/mnist/ for MNIST dataset and http://www.cs.toronto.edu/~kriz/cifar.html for CIFAR-10 dataset.
